# Effects of Arsenic upon Sheep

**Published:** 1843-06

**Authors:** 


*Effects of Arsenic upon Sheep.*—DANGER and FLANDIN, who have performed many experiments with the view of determining the real extent of the poisonous influence manifested by arsenic on wool-covered animals, announce the following as among the latest results obtained by them: A sheep poisoned by a quantity of arsenic inserted under the skin, died in five days. To the last moment it had refused all kinds of food, and the quantity of arsenic in the urine had progressively increased. Another sheep, poisoned by a dose of an ounce of arsenic mixed with a handful of salt (the stomach was probably otherwise empty, though this is not stated), died, as the foregoing, on the fifth day afterwards. It was ill from the moment of having taken the poison, and, like the other sheep, it continued from that time to refuse all nourishment. This last experiment, if performed under the conditions we have above hinted at, would tend strongly to confirm Rognetta's theory that arsenious acid becomes harmless to ruminating animals by being involved in a great quantity of food, and its absorption being thereby hindered. One of the most important deductions drawn by Danger and Flandin from their late experiments is, that the public health is not endangered by the sale of mutton from animals to which arsenic had been some time before administered for the cure of disease: for the presence and continuance of arsenic in the system is readily detected; first, by reason that the animals become ill, however small the quantity of arsenic absorbed; and, secondly, that they have never become well again till the last vestiges of the poison have been eliminated by the kidneys and other excretory organs.

1. The sheep that survived after taking four drachms of arenious acid, having been killed on the thirty-eighth day after, no part of the carcase was found, on the autopsy, to contain a single appreciable trace of arsenic. A dog to which the viscera were given to eat exhibited not the slightest sign of illness, nor could arsenic be detected on analysis either of its fæces or urine; and six persons who partook of the muscular fibre as food lived on it for twelve days without feeling any inconvenience or symptom distinguishing it from meat of other descriptions. 2. The dog that ate the viscera of three poisoned sheep did not experience fatal results, and when killed on the ninth day from the reception of this food, exhibited a healthy internal appearance, without any trace of arsenic whatever, the entire poison having passed off in the urine during the six days immediately following the introduction of the poison into the system. The comparative harmlessness of the poison on the dog, as compared with the sheep, may be accounted for from the far smaller extent (about one-fifth) of the intestinal canal, as well as the much greater muscularity of the tissues connected with the digestive organs of the carnivorous animals: these causes render the digestion, absorption, and the secretions generally much more active in the dog than the sheep.—*Lond. Lancet,* from *Gazette des Hôpitaux.*

